# ADMET-Guided Design and In Silico Planning of Boron Delivery Systems for BNCT: From Transport and Biodistribution to PBPK-Informed Irradiation Windows

**DOI:** 10.3390/molecules31040617

**Published:** 2026-02-10

**Authors:** Karolina Ewa Wójciuk, Emilia Balcer, Łukasz Bartosik, Michał Dorosz, Natalia Knake, Zuzanna Marcinkowska, Emilia Wilińska, Marcin Zieliński

**Affiliations:** 1 Nuclear Facilities Operation Department, National Centre for Nuclear Research, 05-400 Otwock, Polandlukasz.bartosik@ncbj.gov.pl (Ł.B.); michal.dorosz@ncbj.gov.pl (M.D.); natalia.knake@ncbj.gov.pl (N.K.); zuzanna.marcinkowska@ncbj.gov.pl (Z.M.); emilia.wilinska@ncbj.gov.pl (E.W.); 2 National Atomic Energy Agency, 00-400 Warsaw, Poland; 3 Nomaten, National Centre for Nuclear Research, 05-400 Otwock, Poland; marcin.zielinski@ncbj.gov.pl

**Keywords:** boron neutron capture therapy, BNCT, ADMET, physiologically based pharmacokinetic modelling, boron delivery, nanomedicine, metallacarborane, dose planning

## Abstract

BNCT (Boron Neutron Capture Therapy) is a binary radiotherapeutic modality in which high LET (Linear Energy Transfer) particles are generated from ^10^B(n,α)^7^Li reaction, ideally within boron-loaded tumour cells, so the therapeutic outcome depends critically on the pharmacokinetics and biodistribution of boron carriers. In this review, boron-containing agents for BNCT, with a focus on ADMET (absorption, distribution, metabolism, excretion and toxicity) and model-informed design, were examined. Low-MW (low-molecular-weight) compounds, peptide conjugates, polymeric and nanostructured platforms and cell-based vectors were surveyed and how physicochemical properties, transporter engagement and nano–bio interactions govern tumour uptake, subcellular localisation and normal tissue exposure were discussed. A shift from maximising boron content towards optimising exposure profiles using PET (Positron Emission Tomography), PBK (physiologically based pharmacokinetic) modelling and in silico ADMET tools to define irradiation windows was also discussed. Classical agents such as BPA (Boronophenylalanine) and BSH (Sodium Borocaptate) are contrasted with newer polymeric and metallacarborane-based carriers, with attention to brain penetration, endosomal escape, linker stability, biodegradation and elimination routes, as well as platform-specific toxicities. Incontestably, further progress in BNCT will highly depend on integrating imaging-derived kinetics with PBPK-informed dose planning and engineering subcellularly precise yet degradable carriers, and that ADMET-guided design and spatiotemporal coordination are central to achieving reproducible clinical benefit from BNCT’s spatial selectivity.

## 1. Introduction

BNCT (Boron Neutron Capture Therapy) is a binary radiotherapeutic modality [1,2,3] predicated on the nuclear reaction between ^10^B (boron-10) and low-energy thermal neutrons. The interaction produces high LET (Linear Energy Transfer) α-particles and lithium-7 (^7^Li) nuclei with path lengths of approximately 5–9 μm—comparable to a single cell diameter—thus confining cytotoxicity to boron-loaded tumour cells and sparing adjacent healthy tissues. This spatial selectivity, not achievable with conventional external beam radiotherapy, offers advantages in recurrent or otherwise inoperable malignancies [4,5], notably glioblastoma, head-and-neck cancers, and melanoma.

Clinical success depends critically on the ADMET profile of boron-containing agents [6,7]. To achieve selective tumour ablation, approximately 20–35 μg of ^10^B per gram of tumour tissue is required, with a T/N (tumour-to-normal tissue) ratio exceeding ~3 and a T/B (tumour-to-blood) ratio sufficiently high at the time of irradiation [2,8,9]. Accordingly, chemical design, biodistribution, metabolism, and elimination must be co-optimised within the ADMET framework.

### 1.1. Historical Overview and Clinical Progress

Since the early clinical explorations at Brookhaven in the 1950s, several generations of boron delivery agents have emerged. The benchmarks—BPA (Boronophenylalanine) and BSH (Sodium Borocaptate)—remain reference standards owing to known clinical pharmacokinetics and safety, albeit with limited tumour selectivity and suboptimal retention. BPA relies on LAT1 (L-Type Amino Acid Transporter 1) transport and shows heterogeneous uptake; BSH distributes largely extracellularly with minimal active transport [1,10,11,12]. The monograph by Sauerwein et al. (2012) integrated biokinetics with clinical translation and catalysed subsequent rational design [1]. Parallel advances in synthetic boron chemistry—particularly metallacarborane-modified nucleosides and DNA (Deoxyribonucleic Acid)-intercalating constructs—have expanded the scope of agents combining high boron payloads with favourable biophysics [13,14,15].

### 1.2. Emergence of ADMET-Guided Design

Historically, optimisation prioritised bulk boron concentration over PK (pharmacokinetic) metrics. Contemporary programmes integrate quantitative PK, dynamic PET/MRI (Positron Emission Tomography/Magnetic Resonance Imaging)-based imaging, and computation to predict tissue penetration and retention [3,7,14]. For example, nanocarriers—PEGylated (modified with Polyethylene Glycol) liposomes, boronated dendrimers and mesoporous silica nanoparticles—are engineered to exploit the EPR (Enhanced Permeability and Retention) effect, typically improving tumour exposure versus small molecules [3,15,16]. PEGylation prolongs systemic residence and can markedly lengthen apparent half-life relative to free BPA, improving synchronisation between peak tumour concentrations and neutron exposure [15,17].

### 1.3. Molecular Classes of Boron-Containing Agents

Boron agents for BNCT may be grouped into: (1) low-molecular-weight compounds (e.g., BPA, BSH, boronated nucleosides) [10,11,14]; (2) peptide-based conjugates, e.g., EGFR (Epidermal Growth Factor Receptor) or integrin-targeted [14,18,19]; (3) polymeric carriers, e.g., PAMAM (Epidermal Growth Factor Receptor) dendrimers, PEGylated borates [15,16,17]; (4) nanostructured materials, e.g., BN (boron nitride) nanotubes, liposomes, hybrid nanogels; and [20,21,22] (5) bio-cellular delivery systems (e.g., macrophages or stem cells) [18,21,23]. Each class exhibits distinct ADMET trade-offs: low-molecular-weight agents permeate rapidly but clear renally; polymeric/nanoparticulate systems extend circulation and improve EPR-mediated accumulation yet raise questions of biodegradation and long-term safety; cellular carriers provide pathophysiological homing, including into hypoxic regions, at the expense of higher biological variability [14,23,24].

### 1.4. European Contribution

European groups have played a pivotal role in metallacarborane chemistry and BNCT pharmacology. Work led by Polish institutions reported metallacarborane intercalators and boronated nucleosides with balanced lipophilicity (logP ≈ 2–3) and high chemical stability, translating into improved cellular retention in preclinical models [25,26]. Polish investigators have also demonstrated macrophage-mediated delivery of boron carbide nanoparticles, achieving homing into poorly perfused tumour regions and sustained intratumoural presence [24]. Across Europe, teams in Finland and Italy advanced hybrid polymer–silica carriers that enable imaging-guided delivery and controlled release. Collectively, these efforts illustrate a shift from merely maximising bulk boron to ADMET-guided optimisation of exposure, selectivity and subcellular localisation [13,14,15,21,23,24,27]. These advances in boron chemistry are paralleled by the establishment of dedicated BNCT infrastructure at the MARIA research reactor in Poland, enabling preclinical radiobiological studies [28].

### 1.5. Aim and Structure of the Review

This review provides an ADMET-centred analysis of boron-containing compounds and carriers relevant to BNCT, correlating chemical structure with biodistribution, metabolic fate and toxicity and PK parameters: t_1/2_ (half-life), CL (clearance), V_d_ (volume distribution). The review is organised by the five ADMET pillars, each supported by representative data, mechanistic considerations, and literature cross-references.

## 2. Absorption

Efficient absorption and systemic bioavailability are prerequisites for achieving therapeutic intratumoural ^10^B at irradiation. Key determinants include molecular weight, lipophilicity (logP), solubility, ionisation, and membrane permeability. The principal challenge is to maximise tumour selectivity while securing sufficient exposure to deliver high T/B ratios at the time of neutron capture [2,3,7,14,24]. Table 1, summarises key ADMET parameters discussed in this section. Foundational ADMET principles relevant to this section include classical drug-likeness and permeability heuristics, as well as the BCS (Biopharmaceutics Classification System) framework [29,30,31,32]. Transporter effects, which modulate apparent absorption beyond passive diffusion, are reviewed by the International Transporter Consortium and recent clinical DDI (drug–drug interaction) methodology work [33]. Complementary BNCT-focused overviews of boron-agent classes further contextualise absorption behaviour [34,35,36]. A concise platform-level overview of absorption-relevant properties across boron classes is provided in [23]. Additional class-level insights relevant to uptake and formulation are provided by reviews on CA IX (Carbonic Anhydrase IX)-targeted boron scaffolds, single-boron pharmacophores, and sugar-based/PEGylated PET-tracers [19,37,38,39].

### 2.1. Physicochemical Determinants

Key small-molecule carriers such as BPA and BSH exemplify the trade-off between permeability and selectivity. Optimising logP in the range ~2–3 while maintaining aqueous compatibility tends to favour transmembrane passage and endocytic uptake, especially when carborane motifs are balanced by polar linkers [13,14,15,59]. Hydrogen bond donors/acceptors, steric compactness and charge state at physiological pH collectively determine LAT1 engagement and passive diffusion [10].

BPA, an amino-acid analogue, engages LAT1, yet its low passive permeability (logP ≈ −1.2) constrains diffusion [14]. LAT1, known as SLC7A5, is the most investigated Na^+^- and pH-independent membrane transporter based on antiport mechanism, highly expressed in the BBB (Blood–Brain Barrier), cerebral cortex, BRB (Blood-Retina Barrier), testis, placenta, bone narrow and in various types of cancer such as malignant glioma, multiple myeloma, cholangiocarcinoma, lung, bladder, thyroid, prostate, uterine cervical and breast cancer [60,61,62,63,64,65]. BSH (logP ≈ −4.8) is highly hydrophilic, exhibits minimal membrane permeability and undergoes rapid renal elimination, limiting tumour accumulation [11,12]. Incorporation of carborane/metallacarborane motifs increases lipophilicity (often to logP 2–3) and can enhance passive and endocytic uptake without forfeiting aqueous compatibility when combined with polar linkers [13,14,15,66]. Reports of DNA-intercalating metallacarboranes document greater cellular uptake than BPA with sustained intracellular retention in vitro [13,14,67].

### 2.2. Absorptive Pathways

Absorptive pathways comprise active transport (e.g., LAT1 for BPA; nucleoside transporters for boronated nucleosides) and passive diffusion; peptide conjugates exploit receptor-mediated endocytosis (e.g., EGFR, α_v_β_3_–integrin, highly expressed by osteoclasts) [10,14,18]. Nanoparticles and polymers enter predominantly via clathrin/caveolin-mediated endocytosis with subsequent endosomal trafficking; design solutions include pH-labile linkers and membrane-disruptive elements to promote endosomal escape [15,21,22,62]. Cellular vectors such as macrophages or MSCs (mesenchymal stromal cells) function as cellular carriers, traversing barriers and releasing cargo within the tumour microenvironment [23,24].

### 2.3. Quantitative Considerations

Small-molecule BPA typically achieves rapid plasma peaks with short biological half-life, whereas PEGylated liposomes and related platforms prolong systemic exposure through reduced opsonisation and RES uptake [15,68,69]. For example, transferrin-PEG liposomes (tumour-bearing mice) achieved tumour ^10^B ~35.5 µg/g and maintained tumour ^10^B > 30 µg/g for ≥72 h (tumour/plasma ratio 6.0 at 72 h) [11,15,16,17,23,34,35,48,49,50,51,52]. PET using ^18^F-BPA-fructose (^18^F-labelled Boronophenylalanine-fructose, FBPA-Fr) supports clinically relevant tumour-to-normal brain uptake ratios in glioma patients (see Section 3.6), aligning PET readouts with boron analyses from surgical samples [8,9,70].

### 2.4. Strategies to Enhance Absorption

Approaches include: prodrugging to transiently increase lipophilicity; PEGylation to extend circulation and leverage EPR; nano-engineering particle sizes ~50–150 nm for endothelial permeability–stability balance; biological carriers for hypoxia-directed uptake; and co-administration with permeation enhancers to augment transmembrane transport [14,15,21,22,69].

### 2.5. Key Insights

Absorption is governed by a coupling of physicochemical design and biological transport. Optimising logP (~2–3), molecular compactness and transporter/ligand engagement yields superior cellular entry and retention for boron delivery platforms [14,15,24].

## 3. Distribution

PBPK (physiologically based pharmacokinetic) approaches linking time-varying boron concentrations to dose kernels are increasingly used to synchronise irradiation with peak tumour-to-blood ratios [70]. PEGylation reduces opsonisation and reticuloendothelial uptake, prolonging the terminal half-life and enabling more predictable exposure windows [15,69]. Key ADMET parameters discussed in this section are summarised in Table 2. Distribution behaviour is strongly shaped by transporter expression, protein binding and tissue barriers; contemporary ITC (International Transporter Consortium) guidance and transporter-centric clinical designs provide the broader context for interpreting BNCT carriers [33]. For distribution and selectivity across contemporary agent classes, recent BNCT reviews and clinical guidance are informative [34,35,71]. Early animal studies reported in vivo organ distribution and handling of borylated ferrocenium derivatives, providing additional context for BNCT carrier interpretation [72]. Class-level distribution and selectivity considerations are summarised in [23]. Distribution and targeting considerations are further illustrated by carborane-containing polymers, BODIPY (Boron-Dipyrromethene) imaging scaffolds, and organotrifluoroborate conjugates [11,57,73,74,75,76,77].

### 3.1. Pharmacokinetic Determinants and Modelling

**Figure 1:**
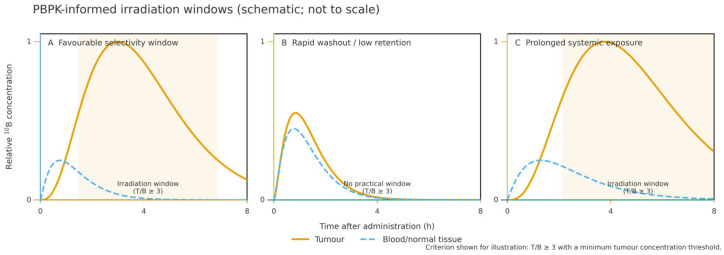
PBPK-informed irradiation windows for BNCT (schematic; not to scale). Illustrative tumour and blood/normal tissue relative 10^10^ of ^10^B concentration–time profiles showing how the usable irradiation window depends on time-varying selectivity (e.g., T/B ≥ 3) and an exposure constraint in blood/normal tissue. (**Panel A**): Favourable window with sustained tumour retention and faster decline in blood/normal tissue. (**Panel B**): Rapid washout and insufficient selectivity. (**Panel C**): Delayed window due to prolonged systemic exposure despite high tumour uptake. Thresholds are illustrative and protocol- and agent-specific (Figure 1 is original artwork created by the authors and does not include third-party copyrighted material; therefore, no copyright permission is required).

Low-molecular-weight agents such as BPA/BSH generally show limited V_d_ (≈0.4–0.6 L/kg), consistent with extracellular confinement and rapid clearance [1,11,12]. Nanocarriers and peptide conjugates often exhibit higher V_d_ (≈1.5–3.5 L/kg), reflecting deeper tissue penetration and longer residence, aided by PEGylation and lipid encapsulation [15,69]. Emerging PBPK and time-dependent boron-dose models seek to harmonise biodistribution with irradiation timing and dosimetry [70]. This concept is illustrated schematically in Figure 1.

Strategies to enhance brain penetration include tuning particle size (50–100 nm), surface charge near neutrality, and grafting ligands for receptor-mediated transcytosis. Nevertheless, clinical translation requires balancing enhanced BBB transport with off-target uptake and potential microglial activation [8,15,23].

### 3.2. Tissue Distribution and Tumour Selectivity

BPA preferentially accumulates in LAT1-expressing tumours but is subject to inter and intratumoural heterogeneity [10]. BSH is comparatively non-selective with higher hepatic/renal uptake [11,12]. Structural targeting (e.g., nuclear-affine metallacarboranes) and ligand-directed systems (e.g., RGD, folate) can increase T/N ratios in preclinical models [13,14,15,21]. Cellular carriers (e.g., macrophages) have delivered more uniform intratumoural distributions, including in hypoxic/necrotic regions [23,24].

### 3.3. Blood–Brain Barrier (BBB) and Blood–Tumour Barrier (BTB)

**Figure 2:**
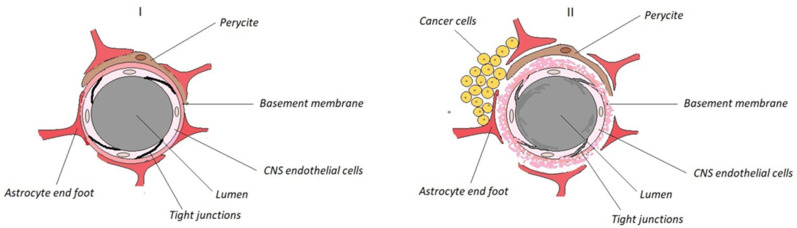
Anatomical structures of Blood–Brain Barrier (**I**) and Blood–Tumour Barrier (**II**).

For primary CNS tumours and metastatic brain tumours, penetration of the BBB/BTB remains a key hurdle. Importantly, primary CNS tumours exhibit different BTB anatomy and permeability compared with metastatic brain tumours arising from peripheral primary cancers such as lung and breast. According to [79] the BTB in primary CNS tumours is generally more permeable than in brain metastases. This phenomenon requires more comprehensive investigation. BBB (I) and BTB (II) anatomical structures are shown in Figure 2.

Studies indicate that BSH does not cross the intact BBB. It is assumed that delivery of BSH to brain tumour cells relies on passive diffusion across the BTB, owing to its pathologically increased permeability compared with the normal BBB structure [80,81,82]. However, BTB permeability is often functionally insufficient for many molecules because BTB disruption is spatially heterogeneous—parts of the tumour and especially infiltrative margins retain BBB-like barrier properties, limiting passive diffusion and leading to uneven delivery [79,80,81,82]. In this review, we consider permeability ‘sufficient’ when it enables BNCT-relevant intratumoural ^10^B levels and selectivity at the planned irradiation time (e.g., adequate tumour uptake with favourable tumour-to-blood/normal ratios) [82].

**Figure 3:**
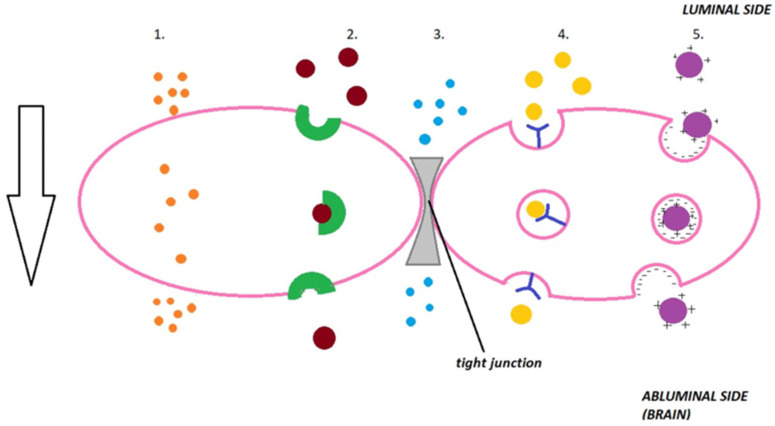
Mechanisms of transport across BBB: (**1**). Transcellular transport lipid soluble molecules (ethanol, steroid hormones); (**2**). Carrier-mediated transport (CMT) of molecules (glucose, amino acids, e.g., BPA via LAT1 transporter, monocarboxylates, nucleosides, small peptides); (**3**). Paracellular transport of water-soluble substrates (through tight junctions); (**4**). Receptor-mediated endocytosis of large molecules (insulin, transferrin, ApoE, leptin, amyloid beta); (**5**). Absorptive-mediated endocytosis of native plasma proteins like albumin [84].

BPA/BPA-fructose partially circumvents the BBB via LAT1 transporters but shows heterogeneous uptake. PEGylated liposomes, boronated polymeric nanoparticles and boron-rich clusters can exploit receptor-mediated transcytosis or adsorptive endocytosis; functionalisation (e.g., transferrin-like ligands) and intermediate lipophilicity can improve brain exposure (predominantly in vivo, preclinical evidence for carrier platforms; clinical evidence exists for BPA/BPA-fructose), although translation into consistent clinical benefit requires further validation [15,23,68,83]. Mechanism of transport across BBB is shown in Figure 3 [84].

Preclinical studies on intracerebral drug delivery to brain tumours by intrathecal injection into the cerebrospinal fluid or convection-enhanced delivery directly to intracranial tumours, as potential methods of BBB/BTB bypass, show enhanced ^10^B cellular uptake in lesion areas and higher T/N ratios [82]. However, these methods are highly invasive, and further work on safer BBB/BTB permeability enhancement is required. BBB/BTB permeability for therapeutic drugs can be modified by osmotic mannitol-induced opening of endothelial tight junctions. Furthermore, stimulation of endothelial cell receptors via intra-arterial administration of vasoactive compounds, ultrasound-based techniques combined with microbubble administration, and electropermeabilisation have all been reported as potential methods for clinical BNCT applications [9,79,82,85,86,87,88,89,90]. Nevertheless, despite their potential benefits, these approaches are generally considered invasive and potentially damaging. Consequently, strategies that exploit physiological BBB transport mechanisms and/or the pathophysiological properties of the BTB are being extensively developed as more advantageous and safer alternatives.

### 3.4. Intracellular Distribution and Organelle Targeting

Given the micrometric range of BNCT particles, subcellular localisation matters. Nuclear-accumulating metallacarborane intercalators and boronated nucleosides enhance DNA-proximate ^10^B deposition, potentially increasing cytotoxic yield at equal bulk boron levels. Liposomal/polymeric carriers may localise to endo-lysosomal compartments unless engineered for triggerable release [13,14,15,25,26,67,91,92].

### 3.5. Distribution Kinetics and Clearance

Small molecules approach distribution equilibrium rapidly (≈15–30 min), whereas nano-systems often show bi-exponential kinetics with prolonged α- and β-phases owing to stealth coatings and reduced RES uptake [15,63]. Biodistribution patterns typically favour tumour and liver for silica/BN-based nanocarriers, with PEG minimising splenic/renal deposition relative to non-PEGylated analogues [15,21].

### 3.6. Imaging-Based Distribution Data

Clinical FBPA-PET studies in glioma demonstrate T/N ratios commonly around 2–3+ with supportive T/B values, guiding patient selection and scheduling [8,40,70]. Accelerator-based BNCT platforms and prospective trials are expanding the clinical dataset and will enable more robust ADMET-dosimetry integration [5,11,24,85].

## 4. Metabolism of Boron-Containing Agents

Successful BNCT requires that chemically stable ^10^B remains associated with tumour cells long enough to match the neutron-irradiation window, while minimising residues in normal organs. Here, “metabolism” encompasses (i) enzymatic and non-enzymatic transformation of small-molecule agents and linkers, (ii) intracellular trafficking and processing that determine subcellular fate, and (iii) biodegradation of carrier matrices and ligands that release, retain, or inactivate boron payloads [1,2,3,7,14,15,76,92,93]. Table 3 provides a concise summary of the key ADMET parameters discussed in this section. In silico ADMET platforms can be used to anticipate metabolic liabilities of linkers and to triage designs prior to in vivo testing [34]. Mechanistic reviews of boron pharmacophores (including carborane-based scaffolds) summarise metabolism-relevant design trade-offs [34,35]. Metabolic stability of boron pharmacophores and linker design considerations are overviewed in [64]. Metabolism-relevant design trade-offs (cluster stability vs. linker liability) and polymer platforms are discussed in recent reviews and case studies [19,39,48].

### 4.1. Low-Molecular-Weight Agents

BPA/BPA–fructose. BPA is transported predominantly by LAT1 and, after cellular entry, does not undergo extensive biotransformation; its apparent “metabolic” behaviour is governed by reversible intracellular partitioning and relatively rapid egress from cells lacking sustained LAT1-mediated transport [10]. Clinically, the fructose complex improves aqueous handling and exposure but does not introduce a distinct metabolic pathway; PET studies with ^18^F-BPA (^18^F-labelled BPA) analogues show time-dependent tumour uptake and washout consistent with transporter-coupled distribution rather than covalent metabolism [8,40]. Consequently, strategies to increase functional retention rely on formulation or co-delivery that delays efflux (Section 4.4) [3,15,17].

BSH. BSH is highly hydrophilic and largely extracellular; it exhibits limited membrane permeability and is cleared renally with negligible metabolic conversion, which constrains intracellular boron residence times in many tumours [1,11,12]. Attempts to alter BSH fate therefore emphasise conjugation or encapsulation rather than exploiting intrinsic biotransformation [3,7,27].

### 4.2. Metallacarborane and Carborane-Containing Small MOLECULES

In this review, the term “metallacarboranes” refers primarily to well-defined transition-metal bis (dicarbollide) boron clusters and their biologically functionalised derivatives, with particular emphasis on nucleoside–metallacarborane conjugates reported as chemically stable, boron-rich constructs and investigated for BNCT-relevant properties. Representative nucleoside–metallacarborane architectures and their structural characterisation are described in refs. [25,26], with additional structure–property discussion for nucleoside/boron-cluster conjugates in ref. [59] and broader metallacarborane medicinal-chemistry context in refs. [13,67]. Carborane clusters are exceptionally resistant to oxidative and hydrolytic degradation under physiological conditions, a property that underpins their use as metabolically robust boron carriers [7,13,14,15,25,26,41]. When appended to nucleosides, intercalators or other scaffolds, metabolic “soft spots” shift from the cluster to organic linkers and heteroatom-rich tethers. Reported designs mitigate premature cleavage through steric shielding, hydrolytically stable linkages, and balanced lipophilicity (logP ≈ 2–3) that reduces lysosomal sequestration without sacrificing uptake [7,13,14,15,25,26,41].

### 4.3. Bioconjugates: Peptides and Targeted Ligands

For peptide or small-protein conjugates, in vivo stability is dominated by proteolysis and linker chemistry. Protease-resistant backbones, cyclisation, and PEGylated spacers reduce proteolytic turnover, whereas cleavable linkers (e.g., acid-labile or enzyme-responsive) can trigger intracellular release from endosomes—minimising lysosomal degradation of the cargo [10,14,15,18,19,91]. Receptor cycling kinetics also shape fate: repeated endocytosis recycling can maintain intracellular exposure if the conjugate avoids rapid lysosomal destruction [14,15].

### 4.4. Polymeric and Lipid Carriers

Polymeric dendrimers and PEGylated liposomes protect boron payloads from premature efflux and metabolism by slowing opsonisation and reticuloendothelial uptake; their effective half-lifes are therefore controlled by colloidal stability, stealth coatings and gradual matrix erosion rather than classical xenobiotic metabolism [15,52,68,69]. PEG corona density, particle size and surface charge govern protein corona formation and downstream clearance; insufficient stealth accelerates hepatic and splenic processing, curtailing the therapeutic window [16,17,56]. Within cells, endosomal–lysosomal trafficking can entrap carriers; endosomolytic components or pH-responsive gates are used to achieve cytosolic release before degradative processing reduces functional boron content [15,16,17].

### 4.5. Inorganic Nanoplatforms

Functionalised mesoporous silica nanoparticles (MSNs) do not undergo enzymatic metabolism; their fate reflects surface chemistry and biodegradation into silicic acid over extended timescales. Gatekeepers and ligand shells dictate when and where boron is released; careful control of linker stability is required to prevent premature shedding in blood or rapid endo-lysosomal degradation after uptake [20,21,53,54,55]. As with polymeric systems, protein corona evolution influences cellular routing and organ-level processing [21,56].

### 4.6. Cell-Based Delivery Systems

Macrophages and related cellular carriers internalise boron payloads and traffic them into hypoxic and poorly perfused tumour regions. In this context, “metabolism” comprises intracellular processing of the payload (e.g., nanoparticle dissolution or linker cleavage) and carrier cell viability and activation state, which together govern release kinetics at the disease site. Available data indicate that macrophage-borne boron carbide nanoparticles can maintain payload integrity during homing and enable sustained intratumoural release (in vitro macrophage loading; in vivo tumour homing/retention), thereby prolonging intratumoural residence without chemical modification of the boron core [23,24].

### 4.7. Analytical Read-Outs and Modelling of Metabolic Fate

Dynamic PET with ^18^F-BPA analogues offers a non-invasive surrogate for time-dependent boron handling in LAT1-positive tissues, informing whether apparent loss reflects distributional washout rather than chemical turnover [8,40,87]. Integrating such data into physiologically based pharmacokinetic and dose-planning frameworks allows explicit scheduling of irradiation to coincide with peak tumour-to-blood ratios and to account for carrier-specific retention versus clearance processes. Contemporary reviews emphasise aligning subcellular localisation with BNCT radiobiology to maximise high-LET yield per retained ^10^B atom [3,14,83].

### 4.8. Design Principles from a Metabolism Perspective

Favour chemically inert boron cores and shift control to linkers and trafficking; tune linkers for on-target release while resisting plasma degradation [7,13,14,15,25,26,41].

Reduce endo-lysosomal loss by incorporating pH-labile or membrane-active features to promote endosomal escape [14,15,16,17].

Exploit transporter biology without creating new liabilities; for LAT1-mediated entry, adjust polarity and sterics to curb rapid efflux while retaining engagement [3,10,14,15,61].

Engineer carrier shells for controlled processing; PEG density, size and surface chemistry govern protein corona formation, RES uptake and intracellular routing [15,16,52,56].

Leverage biological carriers when microenvironmental access is limiting; release kinetics then arise from cellular turnover rather than chemical degradation [23,24].

Couple measurement to planning; use PET-derived kinetics and PBPK-informed scheduling to match irradiation to periods of maximal on-target retention [8,40,70,83,92].

## 5. Excretion of Boron-Containing Agents

The elimination of boron carriers determines the width of the therapeutic window, the off-target dose to radiosensitive organs, and the long-term safety profile. For BNCT agents, “excretion” encompasses glomerular filtration and tubular handling for low-molecular-weight compounds, hepatobiliary elimination following uptake by hepatic sinusoidal endothelium and Kupffer cells, and MPS (mononuclear phagocyte system)/RES processing of nano and biocarriers, often preceded by protein corona formation. Excretion-relevant ADMET parameters across representative agents are summarised in Table 4. Elimination considerations for BPA/BSH and newer classes are discussed in current BNCT guidance and reviews [34,78]. Early animal work on borylated ferrocenium derivatives provides additional context on organ distribution and handling [72]. Elimination routes and practical safety context for boron classes are discussed in [23]. Elimination and material handling can be informed by reports on 2D boron nitride nanosheets and tracer pharmacokinetics [58,95,96].

### 5.1. General Principles and Elimination Pathways

Small, hydrophilic molecules are typically cleared rapidly by the kidneys; increased lipophilicity and plasma protein binding reduce filtration and may favour hepatobiliary routes. For nanoparticles and supramolecular carriers, hydrodynamic size, surface chemistry (including PEG density and charge), and the acquired biomolecular corona govern MPS recognition and organ retention, thereby shaping biliary versus renal elimination and any lymphatic drainage component [49,50,51,96]. In practice, systems that avoid opsonisation and remain below renal filtration size cut-offs are more likely to undergo efficient urinary clearance; larger or poorly “stealthed” constructs preferentially accumulate in liver and spleen before slowing hepatobiliary excretion [49,50,51].

### 5.2. Low-Molecular-Weight Agents

BPA/BPA–fructose. After LAT1-mediated uptake and redistribution, BPA is eliminated predominantly via the kidneys; clinical PET kinetics consistently show relatively rapid blood clearance with time-dependent tumour washout, reflecting transporter biology more than metabolic turnover [3,40,68]. Adjusting infusion schedules and irradiation timing aims to exploit transiently favourable tumour-to-blood ratios while limiting renal exposure [3,14].

BSH. Sodium borocaptate, being highly hydrophilic and largely extracellular, exhibits rapid renal elimination with modest cellular retention; hepatobiliary clearance is limited relative to urinary excretion [1,11,12].

### 5.3. Bioconjugates (Peptides, Targeted Ligands)

Peptide conjugates below the renal filtration size tend to be cleared renally unless protected by plasma binding or sustained receptor engagement; proteolysis generates smaller fragments that further favour urinary elimination. Where conjugates engage high-turnover endocytic pathways, intracellular routing to lysosomes can precede efflux of catabolites and subsequent renal clearance. Linker design (e.g., acid-labile, enzyme-responsive) modulates when intracellular release occurs and may indirectly alter the balance between renal and biliary routes [14,15,18,19,91].

### 5.4. Polymeric and Lipid Carriers

PEGylated liposomes and dendrimers generally exhibit prolonged circulation with progressive uptake by the MPS in liver and spleen; elimination typically proceeds via hepatobiliary excretion of lipidic or polymeric catabolites and, to a lesser extent, renal clearance of low-molecular fragments. Higher PEG density and near-neutral surface charge reduce opsonisation and slow RES capture, whereas insufficient stealth accelerates hepatic processing and shortens systemic exposure [15,16,17,52]. Re-engineering endosomal escape may paradoxically increase apparent elimination if it accelerates cytosolic release and subsequent renal clearance of small payloads [16,17,52].

### 5.5. Inorganic Nanoplatforms

Functionalised mesoporous silica nanoparticles (MSNs) do not undergo enzymatic metabolism; instead, they experience gradual biodegradation to silicic acid, with rates governed by particle size, porosity and surface functionalisation. Depending on corona composition and organ uptake, elimination can involve slow hepatobiliary routes, with urinary excretion of soluble degradation products. Gatekeepers and ligand shells, while enabling on-target release, also influence organ retention and therefore the timescale of excretion [20,21,52,54].

### 5.6. Cell-Based Delivery Systems

For macrophage-mediated delivery, the primary determinant of excretion is the fate of the carrier cell and the persistence of the internalised payload. After tumour homing and release, residual payload may be sequestered by local phagocytes or cleared via lymphatics and hepatic pathways; when cargo consists of inert boron carbide nanoparticles, systemic redistribution is limited and elimination depends on slow biological turnover rather than classical renal or biliary routes.

### 5.7. Transporters and Clinical Pharmacology

Although most BNCT carriers are not classic substrates for drug-metabolising enzymes, renal and hepatic transporters modulate apparent clearance of small molecules and catabolites. Contemporary guidance highlights clinically relevant transporters, e.g., OATs (Organic Anion Transporters)/OCTs (Organic Cation Transporters), OATP (Organic Anion Transporting Polypeptides/BCRP (Breast Cancer Resistance Protein)/P-gp (P-Glycoprotein) and study designs for evaluating transporter-mediated interactions; such principles are valuable when BNCT agents are co-administered with supportive medications that may affect renal secretion or biliary efflux [33,46].

### 5.8. Design Principles for Favourable Elimination

Match elimination route to clinical scheduling: for BPA and similar small molecules, fast renal clearance demands irradiation windows aligned to peak tumour exposure; for nanocarriers, anticipate slower hepatobiliary excretion and plan accordingly. Exploit size and stealth judiciously: maintain hydrodynamic sizes and surface chemistries that avoid excessive RES uptake while not preventing eventual elimination; higher PEG density and neutrality generally prolong circulation but may also delay clearance [16,17,49,50,51].

Design degradability without premature loss: for polymeric and silica systems, incorporate controlled-degradation features that permit eventual elimination (renal or biliary) after the therapeutic window, avoiding long-term organ retention [20,21,52,53,54,55].

Consider transporter context: when feasible, avoid strong interactions with renal/hepatic transporters liable to drug–drug interactions in multi-agent regimens; consult transporter frameworks when planning clinical protocols [33].

## 6. Toxicity and Safety of Boron-Containing Agents

Clinical translation of BNCT depends on delivering a cytocidal high-LET dose selectively to tumour while maintaining acceptable systemic and organ-specific safety. Toxicity arises from (i) the carrier itself (chemical/biological effects), (ii) off-target boron deposition in normal tissues exposed during irradiation, and (iii) platform-specific liabilities (e.g., nanocarrier MPS uptake, peptide immunogenicity) [1,2,3,4,5,7,14,83,93,97]. A compact cross-platform comparison of safety endpoints and mitigations is provided in Table 5. A recent review on boron nanodrugs for BNCT provides an integrated overview of efficacy, biodistribution and safety issues for nano-scale delivery systems [98].

### 6.1. Clinical Safety Experience and Normal Tissue Effects

Modern trials and clinical series, particularly with accelerator-based BNCT for recurrent head-and-neck cancer and other indications, report toxicity profiles dominated by expected radiotherapy-like adverse events in the irradiated field (mucositis, dermatitis, xerostomia), with systemic events generally mild to moderate when BPA or BSH are employed according to contemporary protocols [4,39,83,92,93,97,99]. Dose planning that integrates time-dependent tumour-to-blood ratios reduces normal tissue dose and correlates with acceptable acute and subacute toxicity in prospective cohorts [5,70,83,92,93,99].

### 6.2. Small-Molecule Agents

BPA/BPA–fructose. Clinical use is associated most commonly with transient nausea, flushing and infusion-related symptoms; significant organ toxicity is uncommon at recommended exposures when irradiation is scheduled to favourable T/B windows. Because BPA relies on LAT1-mediated transport, heterogeneity in transporter expression can lead to variable normal tissue uptake, which is mitigated by PET-based selection and scheduling [3,8,10,14,40].

BSH. With its largely extracellular distribution and rapid renal elimination, BSH shows modest tumour selectivity and correspondingly conservative safety margins; organ toxicities are primarily constrained by irradiation geometry and blood concentration at the time of neutron exposure [1,11,40,92].

### 6.3. Bioconjugates and Targeted Ligands

Peptide and ligand-bearing constructs introduce platform risks: proteolysis (yielding reactive fragments), potential immunogenicity, and off-target binding where receptors are expressed on normal tissues. Rational design (protease-resistant backbones, constrained/cyclised scaffolds, stable yet triggerable linkers) reduces these liabilities; nonetheless, receptor density heterogeneity implies that patient selection and imaging confirmation remain crucial to limit unintended normal tissue dose during irradiation [14,15,18,19,91].

### 6.4. Plymeric and Lipid Carriers

PEGylated liposomes and dendrimers prolong circulation and can enhance tumour exposure, but they interact with the MPS, driving hepatic/splenic deposition. Safety considerations therefore include hepatosplenic enlargement, alteration of liver enzymes, complement activation, and—less commonly—infusion reactions; these are mitigated by optimising PEG density, surface charge and size, as well as by step-wise dosing where appropriate [15,16,17,52]. Endosomal escape motifs that improve intracellular delivery must be balanced against membrane-active toxicity at high concentrations.

### 6.5. Inorganic Nanoplatforms

Functionalised mesoporous silica nanoparticles (MSNs) are not metabolised enzymatically but undergo gradual biodegradation to silicic acid, the rate of which depends on particle size, porosity and surface chemistry. Reported safety concerns reflect long-term organ retention if degradation is slow and inflammatory responses modulated by protein corona composition; design strategies that favour biodegradation after the therapeutic window and neutral corona profiles generally improve tolerability [20,21,54,55,56,98].

### 6.6. Cell-Based Delivery Systems

Macrophage-mediated delivery offers deep penetration into hypoxic tumour territories but raises distinct safety questions: persistence of carrier cells, ectopic accumulation, and immune activation. Preclinical work shows tumour homing with sustained intratumoural presence and limited systemic redistribution of inert boron carbide payloads; translation requires GMP (Good Manufacturing Practice)-compliant manufacturing, release criteria, and monitoring for cytokine-related events [23,24].

### 6.7. Radiobiology-Informed Risk Management

Because the path length of α/^7^Li particles is micrometric, toxicity from BNCT is tightly coupled to where boron resides at irradiation. Contemporary radiobiology overviews emphasise aligning subcellular localisation—with a preference for DNA-proximal boron for efficacy—with sparing of normal tissues, especially in organs at risk adjacent to the target volume. PET read-outs (e.g., with ^18^F-BPA analogues) combined with PBPK-aware dose planning reduce normal tissue exposure by matching neutron fields to windows of maximal tumour selectivity [8,40,70,83,92].

### 6.8. Drug–Drug Interactions and Supportive Care

While BNCT agents are not classical substrates for drug-metabolising enzymes, small boronated molecules and catabolites can be substrates of renal or hepatic transporters. Guidance from the International Transporter Consortium and recent clinical methodology highlights the need to consider transporter-mediated interactions when planning antiemetics, analgesics or other supportive medications that might alter renal secretion or biliary efflux [33]. For nanoplatforms and bioconjugates, interactions are more likely to be physicochemical/biological (e.g., complement activation, opsonisation) than enzymatic, and should be addressed through premedication policies and infusion-rate control where indicated.

### 6.9. Practical Design Principles (Safety)

Engineer selectivity first: subcellular localisation and tumour selectivity (T/N, T/B at irradiation) are dominant determinants of toxicity in BNCT; imaging-guided scheduling is central to risk reduction [3,4,5,8,22,40,70,83,93,99].

Minimise pro-inflammatory cues: favour PEGylation and near-neutral surfaces to reduce opsonisation/complement activation for nanoplatforms; avoid highly cationic designs associated with haemolysis or membrane damage [15,16,17].

Implement time-controlled degradability: for inorganic/polymeric carriers, design controlled post-treatment biodegradation, to prevent long-term organ retention while preserving intratumoural residence during therapy [20,21,53,54].

Constrain peptide/ligand risk: use protease-resistant motifs and validate receptor expression in normal tissues to limit off-target uptake; consider immunogenicity screening for repeated dosing [14,15,18,19,91].

Account for transporters and co-medications: map plausible transporter liabilities for small molecules and plan supportive therapy to avoid increasing systemic exposure or altering renal clearance [33].

### 6.10. Genetic and Oxidative Safety

Genetic and oxidative risks associated with boron-containing agents arise from two separable sources: (i) material intrinsic effects (e.g., surface reactivity of inorganic frameworks, redox or photoactive motifs, linker-driven off-target chemistry) and (ii) BNCT irradiation when boron is mislocalised to normal tissues. Contemporary radiobiology underscores that the micrometric range of the α/^7^Li particles confines DNA injury to boron-loaded cells; hence, genotoxicity in healthy tissues is primarily a function of biodistribution at the instant of irradiation, rather than of intrinsic mutagenicity of the carrier [83,92]. For small molecules (BPA/BPA-fructose, BSH), clinical and translational reports have not identified consistent genotoxic signals at therapeutic exposures when irradiation is scheduled to favourable tumour-to-blood ratios and patient selection uses PET read-outs [3,4,5,8,40,92,93]. For supramolecular and inorganic platforms (PEGylated liposomes/dendrimers; mesoporous silica), oxidative read-outs largely reflect protein corona composition, endo-lysosomal residence and long-term organ retention; neutral coronas, controlled post-treatment biodegradation (for silica), and calibrated endosomal-escape features mitigate these risks. Boron nitride nanosheets prepared under sustainable, biocompatible protocols likewise exhibit favourable oxidative profiles contingent on surface passivation and synthesis route [89]. Overall, genetic safety is dictated by selective localisation at the time of neutron exposure and by surface/linker engineering that minimises ROS (Reactive Oxygen Species)-linked stress during residence within the reticuloendothelial system [15,16,17,54,55,56,83,95].

### 6.11. In Vivo Toxicological Profiles and NOAEL (No-Observed-Adverse-Effect Level) Values

For inorganic borates/boric acid outside the BNCT context, classical toxicology synthesises organ-level hazards and NOAEL benchmarks in animals; these reviews provide a conservative backdrop for interpreting systemic exposure (notably renal) and for framing dose-setting in boron-containing platforms [100]. In nanomedicine, clearance-centred analyses relate hydrodynamic size, PEG density, surface charge and corona formation to organ sequestration and elimination, offering practical levers to reduce long-term tissue burden and to interpret hepatic or splenic findings under repeat dosing [49,50,51]. Translational BNCT series indicate that with contemporary BPA/BSH protocols, imaging-guided scheduling and PBPK-aware planning, systemic toxicity is usually limited and dominated by field-restricted radiotherapy reactions rather than multi-organ failure, aligning with clinically acceptable safety margins [4,101]. For mesoporous silica and polymeric carriers, in vivo profiles improve when matrices are degradable on clinically relevant timescales and when surfaces limit opsonisation, thereby facilitating eventual elimination and tempering chronic organ retention [20,21,52,54]. Precise NOAELs are compound-, species- and regimen-dependent; where they exist, they should be invoked in context and not extrapolated across platforms without adjustment for formulation and exposure kinetics [101].

### 6.12. Immunotoxicity and Inflammatory Responses [14,15,16,17,18,20,54,56,91]

Immunotoxicity with BNCT delivery systems is generally platform-determined. For liposomes and dendrimers, principal concerns include complement activation and mononuclear phagocyte system (MPS/RES) uptake, exacerbated by low PEG density, cationic surfaces or “sticky” coronas; mitigation relies on increasing PEG coverage, maintaining near-neutral charge, moderating infusion rates, and using premedication where indicated. For mesoporous silica and related inorganics, inflammatory signatures track with biodegradation kinetics and surface chemistry; designs that promote neutral coronas and triggerable (pH/enzyme-labile) gates tend to suppress innate activation and favour “silent” clearance. Peptide/ligand conjugates carry risks of immunogenicity and off-target binding where receptors are expressed in normal tissues; protease-resistant scaffolds, validated receptor maps and monitoring for infusion reactions are prudent safeguards. Cell-based carriers may elicit cytokine responses contingent on cell activation state and persistence; GMP-compliant manufacture, explicit release criteria, and protocolised clinical monitoring are required. In practice, surface neutrality, controlled degradability and calibrated intracellular escape reduce inflammatory risk, while precise irradiation timing minimises radiogenic inflammation in normal tissues adjacent to the target volume. For a compact cross-platform comparison of safety endpoints and mitigations, see Table 5.

**Table 5:** Toxicity/safety considerations for representative boron-containing agents.

Representative	Principal Toxicity Endpoints	Mechanistic Drivers	Organs at Risk	Mitigation Strategies	Clinical/Preclinical Notes	Quantitative Endpoints Explicitly Reported in Cited Sources	Representative Refs.
BPA/BPA–fructose	Infusion-related symptoms (nausea, flushing); field-limited RT (Radiation Therapy)-like AEs (Adverse Effects) during BNCT (mucositis, dermatitis)	Transporter-driven normal tissue uptake (LAT1); exposure at irradiation if T/B suboptimal	Oral mucosa/skin in field; kidney (exposure during infusion)	PET selection; schedule to peak T/B; supportive care protocols	Systemic toxicity generally mild–moderate at clinical dosing with proper scheduling	500 mg/kg BPA (clinical dosing) reported in clinical studies;.	[3,4,5,8,10,14,23,40,83,93,97,99]
BSH	RT-like AEs in field; limited systemic toxicity	Extracellular distribution; blood concentrations at irradiation	Kidney (rapid renal handling); liver (minor)	Dose planning to minimise normal tissue dose; consider carriers to improve selectivity	Conservative safety margins when scheduling is respected	100 mg/kg BSH (infusion), dose rate 1 mg/kg/min	[1,23,78,92]
Targeted peptides/ligand conjugates	Potential immunogenicity; off-target binding; infusion reactions (rare)	Proteolysis; receptor expression in normal tissues; endosomal trapping	Receptor-positive normal tissues; liver (if opsonised)	Protease-resistant designs; validate receptor maps; premedication/infusion-rate control	Risk profile depends on-target expression and linker chemistry	NR	[14,15,18,19,23,33,37,91,100]
PEGylated liposomes/polymeric dendrimers	Complement activation; hepatic/splenic deposition; infusion reactions	Protein corona→MPS (RES) uptake; insufficient stealth; cationic surfaces	Liver, spleen; blood (infusion)	Increase PEG density; near-neutral charge; graded dosing; endosomolytic features within safe range	Monitoring liver enzymes; mitigate CARPA (Complement Activation-Related Pseudo Allergy)-like events if relevant		[11,15,16,17,23,48,49,50,51,52,56]
Functionalised mesoporous silica nanoparticles (MSNs)	Inflammation with prolonged retention; long-term organ sequestration if slow degradation	Slow biodegradation to silicic acid; corona-modulated responses	Liver, spleen; reticuloendothelial system	Design for controlled post-treatment degradation; neutral corona; dose staggering	Favourable profiles when degradability and surface chemistry are optimised	NR	[20,21,23,53,54,55,56]
Cell-based carriers (e.g., macrophages)	Immune activation/cytokine-related events; ectopic accumulation	Cell persistence/activation state; payload stability	Liver/spleen (clearance); lymph nodes; tumour microenvironment	GMP manufacturing; viability/release criteria; clinical monitoring	Preclinical studies show tumour homing with limited systemic redistribution of inert payloads	Exposure range tested in viability assays: 0.1–200 µg/mL (B4C1/B4C2).	[23,24]
Imaging-oriented boron tracers (e.g., ^18^F-labelled amino acids, sugars)	Low systemic toxicity at tracer doses	Transporter-mediated uptake; rapid clearance	Kidney; field-specific effects not applicable (diagnostic use)	Standard radiotracer safety; QC (Quality Control) of radiochemistry	Useful for selection/scheduling; not therapeutic on their own	NR	[8,38,39,40,48,57]
Historical ferrocene-based boron agents (preclinical)	Organ sequestration-related concerns	Cationic complex organotropism	Liver, spleen, kidney	Preclinical toxicity mapping; not for routine clinical use	Context for organ-level safety considerations	NR	[72]

## 7. Key Insights

### 7.1. Absorption

Transporter biology dominates small-molecule entry: LAT1 for BPA and nucleoside transporters for selected boronated bases, while formulations such as BPA–fructose primarily modulate exposure without creating new biotransformation pathways [8,10,14,23,29,30,32,40]. For nano and polymeric systems, hydrodynamic size (≈50–150 nm), near-neutral surface charge and PEGylation govern epithelial/endothelial passage and endocytic uptake; optimising these variables improves synchrony between tumour exposure and irradiation [11,15,16,17,23,48,49,50,51]. Carborane motifs increase lipophilicity and membrane interaction; balancing logP (~2–3) with polar linkers enhances uptake while mitigating lysosomal sequestration [7,13,14,15,41,42,43,44]. In practice, transporter-aware design combined with pro-retention features (e.g., endosomal escape, linker selection) and PET tracers, where available, allows absorption trajectories to be quantified and scheduled [8,38,39,40,57,58].

### 7.2. Distribution

Clinical distribution is heterogeneous and indication-dependent: BPA shows LAT1-linked tumour uptake with moderate T/N ratios on PET (see Section 3.6), whereas BSH remains largely extracellular with lower selectivity [1,3,8,10,11,12,14,23,34,78]. PEGylated carriers extend circulation but are progressively taken up by MPS organs (liver, spleen); ligand grafting can redirect a fraction towards tumours, whereas BBB entry generally requires specific targeting mechanisms [11,15,16,23,34,35,49,50,51,52,68]. Subcellular localisation is critical given the micrometric range of BNCT particles: DNA-proximal constructs, such as metallacarborane intercalators, can increase radiobiological yield at similar bulk boron levels [13,14,15,41,43,44]. Integration of PBPK-based planning with imaging read-outs allows irradiation to be scheduled at maximal T/B ratios and within platform-specific biodistribution windows [8,40,70,92].

### 7.3. Metabolism

Carborane clusters are metabolically robust; liabilities are shifted to linkers and attached scaffolds, where hydrolysis can be controlled via amide/urea linkages, steric shielding and appropriate lipophilicity [7,13,14,15,41,42,43,44,45]. Polymeric and lipid carriers undergo colloidal processing rather than classical enzymatic metabolism; their fate is dictated by protein corona formation, stealth properties and intracellular routing [11,15,16,17,23,48,49,50,51,56].

MSNs degrade slowly to silicic acid, with surface chemistry and gate design controlling the timing of payload release and material clearance [20,21,54,55]. For BPA/BSH, most apparent “metabolic” behaviour reflects distributional washout, so linker and gate design should align intracellular release with the planned irradiation window [3,8,14,22,37,40,70].

### 7.4. Excretion

Small hydrophilic agents are predominantly cleared renally, whereas nano and polymeric systems tend to follow hepatobiliary routes after MPS uptake, with clearance rates governed by degradability and corona evolution [1,3,11,12,15,23,49,50,51,56]. Controlled degradability of matrices enables eventual elimination while preserving sufficient exposure during therapy [20,21,52,54,55]. Consequently, particle size and stealth should be tuned for predictable clearance, and irradiation should be planned near the pharmacokinetic peak for small molecules or within the exposure plateau for long-circulating carriers [8,40,49,50,51,69].

### 7.5. Toxicity

Most observed clinical toxicities resemble conventional radiotherapy-related effects within the irradiated field; systemic events are generally mild when scheduling aligns with favourable T/B ratios [4,5,83,92,93,97,99]. Platform-specific risks include complement activation (liposomes, dendrimers), inflammatory responses with slowly degradable inorganics, and immunogenicity or off-target binding for peptide/ligand conjugates [14,15,18,20,21,52,53,54,55,91]. These risks can be mitigated by surface neutrality and adequate PEG coverage, controlled biodegradation, validated receptor expression maps and appropriate supportive care protocols, while PET/PBPK-based planning helps minimise normal tissue dose [8,14,15,40,52,54,55,56,69,83,92].

**Figure 4:**
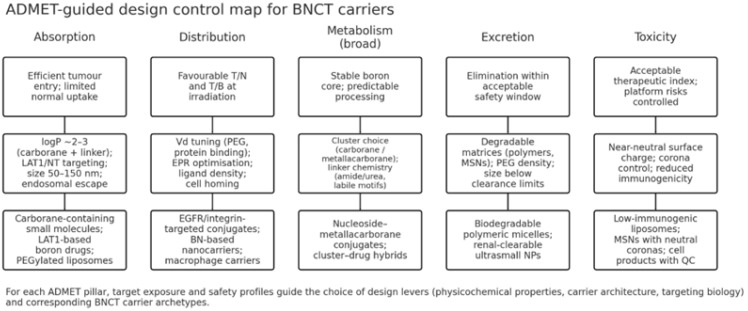
ADMET-guided design control map for BNCT carriers. For each ADMET pillar (absorption, distribution, metabolism, excretion and toxicity), target exposure and safety profiles are linked to concrete design levers (physicochemical properties, carrier architecture and targeting biology) and to representative BNCT carrier archetypes (small molecules, peptide/ligand conjugates, polymeric and inorganic nanocarriers and cell-based delivery systems) (Figure 4 is original artwork created by the authors and does not include third-party copyrighted material; therefore, no copyright permission is required).

These key insights can be mapped back onto the design control framework outlined in Figure 4, emphasising how quantitative ADMET goals translate into concrete choices of BNCT carrier platforms.

## 8. Conclusions and Outlook

Structure–performance relationship and practical implications. For BNCT, the most useful structure–performance relationship is the link between molecular structure (or carrier architecture) and PK/BD, because efficacy depends on delivering sufficient ^10^B to tumour tissue within the irradiation window while limiting normal tissue exposure [1,7,14,15].

BPA (commonly formulated as BPA–fructose) remains the principal clinical benchmark due to transporter-associated tumour uptake and the availability of human PK models used to support scheduling [8,40,62,63,95]. BSH is a complementary reference compound: it is chemically robust and strongly hydrophilic, which favours rapid clearance and largely extracellular distribution, but typically provides limited tumour selectivity unless delivery conditions are optimised [1,9,78,81].

Boron clusters (carboranes and metallacarboranes) offer high boron content and metabolic stability; therefore, performance is often determined by substituents and linkers that control charge, lipophilicity and intracellular retention [13,41,59]. Nucleoside–metallacarborane conjugates provide well-defined examples of this design space and have been structurally characterised in the cited primary literature [25,26], with broader context discussed in recent reviews [13,61]. DNA-associated boron constructs have also been explored as a strategy to influence microdistribution at the cellular level, but their development ultimately hinges on in vivo PK/BD and clearance [14,66].

For liposomes, micelles, dendrimers and inorganic carriers, “structure” is best captured by architectural descriptors (size, surface chemistry/PEGylation/ligands, stability and payload form), which govern circulation time, RES uptake, tumour penetration and clearance [15,16,56]. Representative examples discussed here include transferrin-modified PEG liposomes for boron payload delivery [17], core-polymerised boron-conjugated micelles with engineered pharmacokinetics [83], and macrophage-mediated transport of boron carbide nanoparticles to poorly perfused tumour regions [24].

Taken together, the most consistent interpretation is obtained when delivery concepts are supported by in vivo PK/BD (and, where relevant, imaging) and when clearance is characterised on clinically compatible timescales [15,16,39,58,70]. Approaches supported mainly by in vitro uptake or associated with persistent organ retention should be treated as preliminary until in vivo biodistribution, clearance and reproducibility are established [15,16,56].

Dimodality of BNCT and interdisciplinary collaborative research imply that the development in the multidisciplinary scientific areas and evaluation of new technologies is in demand. To achieve high effectiveness of BNCT, an adequate therapeutic dose delivery is desired. The ADMET framework allows defining input parameters for treatment plan establishment and BNCT implementation. Quantitative theranostics approach, subcellular precision of targeting molecules providing long-circulating yet degradable platforms design, biological vectors evaluation, and transporter-aware pharmacology are the main pillars indicating future directions of BNCT development.

### 8.1. Future Directions

*Quantitative theranostics.* Systematically combined PET-derived kinetics (e.g., ^18^F-labelled amino acids and sugars) with PBPK models and time-resolved dosimetry to personalise neutron field timing and reduce the risk of normal tissue exposure [8,38,39,40,57,58,70,92]. Table 6 summarises the in silico toolbox that underpins ADMET-guided optimisation of BNCT agents and treatment schedules.

*Subcellular precision.* Further develop nuclear-affine boron pharmacophores and triggerable gates to maximise DNA-proximate ^10^B at a given total boron load, with an emphasis on linker chemistries that couple endosomal escape to controlled intracellular release [13,14,15,41,43,44].

*Long-circulating yet degradable platforms.* Design long-circulating carriers with well-characterised biodegradation profiles to balance exposure and clearance, and to define post-treatment degradation timeframes that avoid chronic organ retention [11,15,48,49,50,51,52,53,54,55,68,69].

*Biological vectors.* Cell-based delivery under GMP conditions with robust release criteria and clinical monitoring frameworks to exploit homing into hypoxic tumour regions while maintaining safety [23,24].

*Transporter-aware pharmacology.* Map LAT1 and other clinically relevant transporters across indications to stratify patients and to anticipate drug–drug interactions affecting renal and hepatic transport [10,14,33,61].

### 8.2. Perspective

Research on BNCT is conducted at the intersection of radiobiology, medical chemistry, medical physics, medicine, dosimetry and nuclear engineering. The main challenge is spatiotemporal correlation of boron dose deposition, namely high-LET dose delivery within the therapeutic window in which boron is concentrated at the specified subcellular location within the tumour but it hardly ever accumulates in normal tissues. The ADMET framework clarifies chemical and biophysical therapy pillars providing a basis for treatment plan establishment and adequate physical dose deposition in target volume to achieve an expected therapeutic effect and minimise side effects: transporter engagement and formulation (absorption), PBPK-synchronised tissue exposure (distribution), linker- and route-of-entry control (metabolism), degradability-tuned clearance (excretion), and platform-aware risk management (toxicity). Progress over the next few years is likely to be driven by imaging-guided treatment planning evaluation, specific subcellularly targeting carriers and materials that combine prolonged circulation with predictable degradation, enabling BNCT to deliver its spatial therapeutic selectivity with reproducible clinical benefit.

## Figures and Tables

**Table 1:** ADMET parameters related to absorption of representative boron-containing compounds.

Representative	Class/Format	Absorption Determinants	Principal Uptake Pathway	Absorption- Enhancing Strategies	Key Caveats (Absorption)	Quantitative Endpoints Explicitly Reported in Cited Sources	Representative Refs.
BPA/BPA–fructose (Boronophenylalanine–fructose, BPA-Fr)	Low-MW (low-molecular-weight) amino-acid analogue	Hydrophilicity; LAT1 engagement; formulation (fructose)	Carrier (LAT1) ±limited diffusion	Transporter targeting; clinical formulation (BPA-fructose)	Heterogeneous uptake across tumours	logP ≈ −1.2	[8,10,14,23,29,31,32,40]
BSH	Low-MW polyhydroborate	Extreme hydrophilicity; minimal permeability	Primarily extracellular	High-dose/ infusion; carrier-assisted approaches	Rapid renal clearance; modest selectivity	logP ≈ −4.8	[1,3,23]
Metallacarborane- modified nucleosides/DNA-affine constructs	Small molecules with carborane clusters	Moderate logP (~2–3); compactness; linker stability	Passive uptake; endocytic contributions	Balance polarity; endosomal- escape motifs	Lysosomal trapping if over- hydrophobic	NR (Not reported in this review)– numeric values depend on the specific derivative	[7,13,14,15,23,41,42,43,44]
Peptide/ ligand-targeted conjugates, e.g., RGD (Arginine-Glicine Aspartate), EGFR	Targeted bioconjugates	Affinity/avidity; receptor density; linker stability	Receptor- mediated endocytosis	Valency optimisation; protease-resistant backbones	Variable receptor expression; endosomal sequestration	NR (reported endpoints differ strongly between systems)	[14,18,19,23,33,37,45,46,47]
PEGylated boronated liposomes/ dendrimers	Polymeric/lipid nanocarriers (≈50–150 nm)	Size; PEG stealth; near-neutral charge	Endocytosis; EPR-mediated tissue entry	PEGylation; size tuning; long-circulating designs	RES (Reticuloendothelial System) uptake if insufficient stealth	NR (size/Zeta potential are study-specific and reported in individual platform papers)	[2,11,16,17,23,29,30,31,32,48,49,50,51,52]
Functionalised mesoporous silica nanoparticles	Inorganic nanocarriers	Pore/ligand functionalisation; size/shape	Clathrin/caveolin-mediated endocytosis	Ligand grafting; pH-labile gates	Biodegradation timescale context- dependent	NR (platform-specific)	[20,21,23,53,54,55,56]
Cell-based delivery (e.g., macrophages)	Cellular carriers	Cell homing; payload loading	Active trafficking into tumour microenvironments	Optimise loading/release; exploit chemotaxis	Biological variability	B4C preparations used for loading: 32 ± 10 nm (B4C1) and 80 ± 30 nm (B4C2)	[23,24]
Selected PET- oriented tracers (boronated amino acids, sugars)	Low-MW tracers (diagnostic)	Transporter targeting; radiolabelling	Carrier-mediated uptake (LAT1, sugar transporters)	PEGylation/ sugar conjugation for uptake/PK	Translation to therapy requires exposure matching	NR (numeric tracer endpoints are reported in the individual PET papers)	[38,39,57,58]

**Table 2:** ADMET Parameters Related to Distribution of Representative Boron-Containing Compounds.

Representative (Example)	Distribution Determinants	Typical Biodistribution Pattern	Selectivity (T/N; T/B)	BBB/Organ Targeting	Distribution- Enhancing Strategies	Quantitative Endpoints Explicitly Reported in Cited Sources	Representative Refs.
BPA/ BPA-fructose	LAT1 density; hydrophilicity; short t_1/2_	Tumour uptake in LAT1-high tissues; low V_d_	Glioma PET ~2–3+ (context- dependent)	Partial BBB via LAT1	Timing vs. irradiation; formulation	Human (melanoma patients): peak blood 9.4 ± 2.6 µg ^10^B/g at end of infusion; blood clearance t_1/2_ 2.8 h and 9.2 h; skin-to-blood 1.31 ± 0.22 (first 6 h); tumour-to-blood 3.40 ± 0.83 (resected tumours)	[8,10,14,23,34,40,78]
BSH	Hydrophilicity; extracellular confinement	Blood/kidney/ liver; modest tumour deposition	Lower than BPA	Poor BBB penetration	Carrier- assisted delivery	NR (classical PK/BD (pharmacokinetic and biodistribution) values not consistently reported across cited sources)	[1,23,34,78]
Metallacarborane/ DNA-affine constructs	Lipophilicity; nuclear affinity; linker routing	Enhanced cellular/nuclear localisation	Improved local (organelle) targeting	BBB depends on scaffold	Endosomal- escape/linker tuning	NR (quantitative BD not uniform; depends on specific conjugate)	[13,14,15,23,41,43,44]
Targeted peptides/ ligands	Receptor density; valency; stability	Receptor- positive tumour deposition; off-target varies	Higher apparent selectivity with high receptor expression	Transcytosis possible with ligands	Ligand grafting; protease resistance	NR (endpoints platform-specific; report when available in the primary paper)	[14,18,19,23,33,34,35,37,47,78]
PEGylated liposomes/ dendrimers	PEG stealth; size/charge; corona	Tumour + liver/spleen; prolonged circulation	EPR-driven (model-dependent)	BBB limited; ligand- enhanced entry	Stealth; size tuning; long-circulating designs	Transferrin-PEG liposomes (tumour-bearing mice): tumour ^10^B ~35.5 µg/g; tumour ^10^B >30 µg/g for ≥72 h; tumour/plasma ratio 6.0 at 72 h	[11,15,16,17,23,34,35,48,49,50,51,52]
Functionalised MSNs (Mesoporous Silica Nanoparticles)	Surface chemistry; porosity; corona	Tumour (EPR) and liver/spleen	Improved with targeting ligands	BBB limited; ligand-mediated routes	Ligand grafting; neutral corona design	NR (platform-specific)	[20,21,23,34,53,54,55,78]
Cell-based carriers	Homing to hypoxia/ inflammation; cell kinetics	Uniform intratumoural distribution incl. hypoxic zones	Favourable functional selectivity	Cells traverse barriers	Preconditioning; loading optimisation	NR	[23,24,34]
Borylated ferrocenium (animal data)	Organotropism of cationic complexes	Liver/spleen/ kidney predominant sinks	—	—	—	NR	[72]

**Table 3:** ADMET parameters related to metabolism of representative boron-containing compounds.

Representative (Example)	Metabolic Liability/Processing	Intracellular Fate & Trafficking	Linker Chemistry/Trigger	Stability-/Release-Enhancing Strategies	Key Caveats (Metabolism)	Representative Refs.
BPA/ BPA-fructose	Minimal biotransformation; transporter-driven behaviour	Cytosolic pool; relatively rapid egress without sustained LAT1	—	Formulation and scheduling to delay efflux	Heterogeneous LAT1; rapid washout	[8,10,14,23,40]
BSH	Negligible conversion; renal elimination	Largely extracellular	—	Encapsulation/conjugation	Limited cell entry	[1,3,9,23,78,81]
Metallacarborane/DNA-affine	Carborane inert; linker is liability	Risk of endo-lysosomal trapping; possible nuclear localisation	Stable amide/urea; steric shielding	Balance logP; add endosomal-escape motifs	Over-hydrophobicity→sequestration	[13,14,15,23,41,42,43,44,94]
Peptide/ligand conjugates	Proteolysis; endolysosomal degradation	Endocytosis; recycling vs. degradation	Protease-resistant backbones; cleavable linkers	Cyclisation; PEG spacers; valency tuning	Premature plasma cleavage	[14,15,18,19,23,33,47,91]
PEGylated liposomes/dendrimers	Colloidal stability and corona drive fate; limited enzyme metabolism	Endosomal-lysosomal routing unless engineered	pH-responsive gates; cleavable spacers	Increase stealth; tune size/charge; endosomolytic features	RES processing if insufficient stealth	[11,15,16,17,23,48,49,50,51,52,56]
Functionalised MSNs	Biodegradation to silicic acid; corona-driven processing	Lysosomal residence if ungated	pH/enzyme-labile gatekeepers; ligand shells	Surface chemistry control; triggerable gates	Long-term retention if slow degradation	[20,21,23,53,54,55,56]
Cell-based carriers	Cellular processing of payload; no chemical metabolism of boron core	Deep tumour homing; sustained presence	Payload-specific	Optimise loading/release; preserve viability	Biological variability	[23,24]

**Table 4:** ADMET parameters related to excretion of representative boron-containing compounds.

Representative	Primary Elimination Route(s)	Determinants of Clearance	Organ Retention/Sinks	Excretion-Optimising Strategies	Key Caveats	Quantitative Endpoints Explicitly Reported in Cited Sources	Representative Refs.
BPA/ BPA-fructose	Renal (filtration)	Hydrophilicity; transporter-mediated tissue egress	Kidney exposure during infusion; transient tumour retention	Schedule vs. tumour peak; delay efflux where feasible	Rapid washout in LAT1-heterogeneous tumours	t_½_ (blood clearance, biphasic): 2.8 h & 9.2 h	[3,8,23,30,31,32,40]
BSH	Renal (rapid)	Extreme hydrophilicity; poor cell entry	Kidney; minimal tumour residence	Encapsulation/conjugation	High dosing without carriers	NR	[1,3,23]
Peptide/ligand conjugates	Renal for small conjugates/catabolites; hepatobiliary if plasma-bound	Proteolysis; linker stability; receptor cycling	Lysosomes; liver (if opsonised)	Protease-resistant designs; tuned cleavable linkers	Premature cleavage in plasma	NR	[11,14,15,18,23,33,47,91]
PEGylated liposomes/dendrimers	Predominantly hepatobiliary; renal for fragments	PEG density; size/charge; protein corona	Liver, spleen (MPS/RES)	Increase stealth; degradable matrices	Long-term retention if non-degradable	NR	[2,11,16,17,23,48,49,50,51]
Functionalised MSNs	Hepatobiliary (slow); urinary for soluble products	Size/porosity; surface chemistry; corona; biodegradation	Liver/spleen; gradual degradation to silicic acid	Gatekeepers/ ligands; design for biodegradation	Clearance timescale context-dependent	NR	[20,21,23,53,54,55,56]
Cell-based carriers	Biological turnover; lymphatic/hepatic routes	Carrier viability; payload stability	Tumour phagocytes; lymph nodes; liver	Optimise loading/release; ensure viability	Biological variability; regulatory complexity	NR	[23,24]
Historical organ distribution example (ferrocenium derivatives)	Mixed; organ sequestration→slow clearance	Cationic complex behaviour	Liver/spleen/kidney predominant sinks	—	Preclinical context	NR	[72]

**Table 6:** In silico toolbox for ADMET-guided BNCT (overview).

Tool/Framework	Primary Purpose	Typical Inputs	Key Outputs for BNCT	Use Case in this Review	Representative Refs.
Drug-likeness/BCS rules (RO5—Lipinski’s Rule of five; Veber; BCS)	Rapid prescreen of solubility/permeability risk and formulation needs	Calculated physicochemical properties; class-based thresholds	Risk flags for absorption limits; oral bioavailability heuristics	Prioritise linker/scaffold variants for small boron agents	[29,30,31]
ADMETlab-style prediction (ADMETlab 3.0)	Batch prediction of ADMET surrogates to rank candidates	SMILES/structure; descriptor set	Absorption/distribution/toxicity descriptors; comparative scores	Side-by-side evaluation of linker placements and polarity tuning	[102]
Transporter-aware modelling (LAT1 focus)	Assess transporter contribution vs. passive permeation	Docking/LB (Ligand Binding) models; ionisation; permeability estimates	Uptake likelihood via LAT1; interaction risk with transporters	Classify agents as transporter-dominant vs. permeation-feasible	[10,14,33,47,61]
PET-informed PBPK	Time-aligned exposure modelling and irradiation scheduling	^18^F-BPA/sugar PET kinetics; plasma/biopsy boron; physiological priors	Tumour-to-blood trajectories; schedule windows; sensitivity analyses	Place neutron exposure at peak/plateau selectivity	[8,40,70,92]
Nano-clearance modelling (MPS/biodegradation)	Anticipate organ retention and elimination for carriers	Size/charge/PEG density; corona data; degradability parameters	Hepatobiliary vs. renal balance; residence times; risk flags	Balance exposure with clearance; design degradability “timers”	[11,15,16,17,48,49,50,52,53,54,55,56]

## Data Availability

No new data were created or analyzed in this study. Data sharing is not applicable to this.

## Abbreviations

The following abbreviations are used in this manuscript:

ADMET	Absorption, Distribution, Metabolism, Excretion and Toxicity
AEs	Adverse Effects
BBB	Blood–Brain Barrier
BCRP	Breast Cancer Resistance Protein
BN	Boron Nitride
BNCT	Boron Neutron Capture Therapy
BODIPY	Boron-Dipyrromethene
BPA	p-Boronophenylalanine
BPA-fructose	Boronophenylalanine-fructose, BPA-Fr
^18^F-BPA	^18^F-labelled Boronophenylalanine, FBPA
^18^F-BPA-fructose	^18^F-labelled Boronophenylalanine-fructose, FBPA-Fr
BCS	Biopharmaceutics Classification System
BRB	Blood–Retina Barrier
BSH	Sodium Mercaptoundecahydro-closo-Dodecaborate (Sodium Borocaptate)
BTB	Blood–Tumour Barrier
CA IX	Carbonic Anhydrase IX
CARPA	Complement Activation-Related PseudoAllergy
CL	Clearance
CNS	Central Nervous System
DDI	Drug–Drug Interaction
DNA	Deoxyribonucleic Acid
EPR	Enhanced Permeability and Retention (effect)
EGFR	Epidermal Growth Factor Receptor
GMP	Good Manufacturing Practice
ITC	International Transporter Consortium
LAT1	L-Type Amino Acid Transporter 1, SLC7A5
LET	Linear Energy Transfer
LB models	Ligand Binding models
logP	Logarithm of the Partition Coefficient (octanol/water)
Low-MW	Low-Molecular-Weight
MPS	Mononuclear Phagocyte System
MRI	Magnetic Resonance Imaging
MSCs	Mesenchymal Stromal Cells
MSNs	Mesoporous Silica Nanoparticles
NOAEL	No-Observed-Adverse-Effect Level
NR	Not reported in this review
OATs	Organic Anion Transporters
OATPs	Organic Anion Transporting Polypeptides
OCTs	Organic Cation Transporters
PAMAM	Polyamidoamine
PBPK	Physiologically Based Pharmacokinetic
PK	Pharmacokinetic
PK/BD	Pharmacokinetic and biodistribution
PEG	Polyethylene Glycol
PEGylated	Modified with Polyethylene Glycol
PET	Positron Emission Tomography
PET/MRI	Positron Emission Tomography/Magnetic Resonance Imaging
P-gp	P-Glycoprotein
QC	Quality Control
RES	Reticuloendothelial System
RGD	Arginine–Glycine–Aspartate (integrin-binding motif)
RO5	Lipinski’s Rule of five
ROS	Reactive Oxygen Species
RT-like	Radiation Therapy-like
t_1/2_	half-life
T/B	Tumour-to-Blood Ratio
T/N	Tumour-to-Normal Tissue Ratio
V_d_	Volume of Distribution
